# Towards cardiovascular disease prevention in Nigeria: A mixed method study of how adolescents and young adults in a university setting perceive cardiovascular disease and risk factors

**DOI:** 10.4102/phcfm.v13i1.2200

**Published:** 2021-04-07

**Authors:** Nse A. Odunaiya, Temilade B. Adesanya, Emmanuel C. Okoye, Oluwafemi O. Oguntibeju

**Affiliations:** 1 Department of Physiotherapy, Faculty of Clinical Sciences, College of Medicine, University of Ibadan, Oyo State, Nigeria; 2 Department of Medical Rehabilitation, Faculty of Health Sciences and Technology, Nnamdi Azikiwe University, Awka, Nigeria; 3 Phytomedicine and Phytochemistry Group, Oxidative Stress Research Centre, Department of Biomedical Sciences, Faculty of Health And Wellness Sciences, Cape Peninsula University of Technology, Bellville, South Africa

**Keywords:** knowledge, perception, cardiovascular disease, risk factors, adolescents, young adults

## Abstract

**Background:**

Cardiovascular disease (CVD) is a global problem but its increasing prevalence in the working age group in developing countries like Nigeria is concerning and needs urgent attention.

**Methods:**

The study was a mixed method design: quantitative phase with 402 participants and qualitative phase with 16 participants in two focus groups. The participants in the quantitative survey phase completed two questionnaires on the knowledge and perception of CVD and its risk factors. Data from the quantitative cross-sectional survey were analysed using descriptive and inferential statistics. The qualitative data were analysed using content thematic analysis.

**Results:**

We report that 39.1% of the participants had high knowledge whilst 61.9% had low and average knowledge of CVD and its risk factors. Of the participants, 78.1% had a wrong perception of CVD and its risk factors. Participants from faculties of veterinary medicine and basic medical sciences had better knowledge than others who were not medically inclined (*F* = 16.11; *p* < 0.001). Only participants from the faculty of veterinary medicine had the right perception of CVD and its risk factors. There was no significant difference in knowledge and perception scores between male and female participants. The qualitative study buttressed the results from the cross-sectional survey, where adolescents and young adults highlighted academic stress and poverty as major risk factors for CVD.

**Conclusion:**

Adolescents and young adults in this study did not have good knowledge of CVD and its risk factors. They also had a wrong perception about CVD and its risk factors.

## Background

Cardiovascular disease (CVD) is an important public health concern and one of the leading causes of mortality.^1^ It includes numerous conditions such as atherosclerosis, stroke, heart failure, arrhythmia, heart valve problems, angina and cardiomyopathies.^2^ In 2013, CVD-related death contributed to 38% of all non-communicable disease-related deaths in Africa, which is double the number since 1990. This increase could be linked to an increase in population, urbanisation, lifestyle changes and high prevalence of risk factors,^3^ particularly modifiable risk factors such as physical inactivity, smoking, unhealthy diet, hyperlipidaemia, excessive alcohol intake and obesity.^4^

There has been a rise in CVD and its risk factors in developing countries, with high mortality rate amongst younger people, than in developed countries. This can be attributed to the lack of knowledge and effective preventive strategies, which can also be linked to a high rate of poverty in these countries.^5,6,7^ Adequate knowledge of CVD risk factors is the first step towards an effective preventive mechanism against the burden of CVD amongst any population.^8^ Studies have identified children, adolescents and young adults as the target population for prevention program.^5,9^

Adolescence and young adulthood are critical developmental periods characterised by distinct physical, psychological, cognitive and social changes.^9^ They develop a new cognitive capacity, become increasingly independent from their families, are influenced by peers and become involved in new behaviours and responsibilities during the transition into adulthood. Their behaviours can have a significant impact on their current and future health.^9^ Hence, health behaviours are particularly important during young adulthood.^10^ Inasmuch as manifestation of CVD mostly occurs during adulthood, risk factors such as hypertension, obesity, diabetes, unhealthy diet habits, tendency to smoke and others are often present during adolescence and persist into adulthood.^11,12^ Furthermore, development of healthy lifestyles during early adulthood plays a key role in the reduction of CVD risk as these lifestyles are not easily forgone but are usually maintained up to an elderly age.^12^ Because the modifiable risk factors can be prevented, treated and controlled, there is therefore a need for early detection of risk factors amongst adolescents and young adults.^5^ However, this can best be achieved through adequate health information and knowledge of the disease.

Health information, knowledge and perception about diseases and body functions are evident determinants of health status and outcomes.^13,14^ Knowledge and perception has a key role in healthy behaviour and reduction of risk factors as they are essential in making informed decisions and guiding an individual’s decision about health.^8,14^ The role of knowledge has been proposed by several models including the health belief model. This model explains that knowledge of a disease condition influences patient’s attitude and practice, improves compliance to treatments and leads to reduction in prevalence and aversion of complications.^7^ Several studies have been conducted on knowledge, awareness, perception, attitude and risk stratification of CVD and its risk factors in various parts of the world.^15,16,17^ However, there is a paucity of information on the knowledge and perception of CVD and its risk factors amongst adolescents and young adults in Nigeria, and only a few studies have been conducted amongst adults in Nigeria.^8,18^ This paucity of information constitutes a barrier to effective implementation of a prevention programme amongst this subpopulation. This study was therefore aimed at determining the knowledge and perception of CVD and its risk factors amongst adolescents and young adults starting from a university setting.

## Methods

### Study design

The study used a mixed method design, utilising a cross-sectional survey and exploratory qualitative study (focus group discussion).

### Participants

The participants of this study were undergraduates that were randomly selected from the following faculties at the University of Ibadan: art, basic medical science, law, renewable natural resources, science, social sciences and veterinary medicine. Each participant must have spent at least one academic session at the University of Ibadan. Students who had been diagnosed previously with a CVD or currently had a CVD were excluded from this study. A participant who was diagnosed with CVD during this study had an undue advantage over the others.

### Sampling and sample size calculation

The seven faculties were selected from a total of 14 faculties by simple random sampling technique. The participants for the cross-sectional survey were consecutively recruited into the study, whilst those for the qualitative study were purposively selected. The sample size for the cross-sectional survey was calculated using Slovin’s formula, *n* = *N*/1+*N*(e^2^), where *n* = sample size, *N* = total population of the University of Ibadan undergraduates (15 000) and *e* = confidence level = 0.05. A minimum sample size of 387 was deemed fit for the observational study whilst 16 participants were purposively recruited for the focus group discussion. In order to take care of non-response, 433 participants were recruited for the cross-sectional survey.

### Instruments

#### Heart Disease Fact Questionnaire

This is a 25-item instrument that measures knowledge of major risk factors that cause the development of CVD.^19^ Each item on this scale has responses of true, false or I don’t know. Good internal consistency (Kuder-Richardson *r* = 0.77) was demonstrated in a group of 524 ethnically diverse adults with diabetes and excellent discriminant validity. Test–retest reliability was 0.89. Total scale scores were calculated by summing the total number of correct answers. Scores ranged from 0 to 25 and can be converted to percentage. Higher scores indicated higher level of knowledge;^19^ scores of 0–12 indicated low knowledge level, scores of 13–17 indicated average knowledge and scores of 18–25 indicated high knowledge.

This instrument was used to assess the knowledge of the participants about CVD and its risk factors. The questionnaire was self-administered. Correct knowledge of CVD and its risk factors was calculated for each participant using the scoring formula of the questionnaire to determine if the knowledge of the participants about the subject under consideration was high, average or low.

#### The Perception of Risk of Heart Disease Scale

This is a 20-item instrument developed to measure an individual’s perception of the probability of developing heart disease.^20^ Initial testing with a primary care sample of 295 persons > 15 years of age without heart disease demonstrated an internal consistency of 0.68–0.80. Total scale alpha was 80. Test–retest reliability was 0.61–0.76. Construct validity was demonstrated by achieving a significant correlation between the Perception of Risk of Heart Disease Scale and the Health Promotion Lifestyle Profile II (*r* = 0.20–0.39, *p* < 0.01). Each item on the scale is on a 4-point Likert scale with scores ranging from 1 to 4, with 1 being strongly agree and 4 being strongly disagree. Reverse scoring was used for some items (6, 10, 11, 12, 13, 14, 15, 16, 17, 18, 19, 20). Item scores may be summed up as a subscale, followed by total scale score. A higher score on the overall indicates increased perception of risk:^20^ scores of 50–80 indicated positive perception whilst scores of 0–49 indicated negative perception.

This instrument was used to assess the perception of CVD and its risk factors amongst participants, and it was self-administered. Correct answers to questions indicated a positive perception and wrong answers indicated a negative perception. Using the scoring of the questionnaire, a participant’s perception could be assessed.

### The cross-sectional survey

The Heart Disease Fact Questionnaire and the Perception of Risk of Heart Disease Scale were self-administered to the participants. The socio-demographic characteristics, such as age, sex, department, faculty, level of study and marital status, of the participants were also recorded on a biodata form.

### Focus group discussion

This qualitative aspect of the study involved 16 participants from the seven faculties (with two to three participants from each faculty). Each faculty had a minimum of two participants, whilst two faculties had three participants, hence a total of 16 participants. There were two focus groups, with six participants in the first group and 10 in the second group. Other personnel present in the focus group discussion were one of the authors, a moderator and a note taker. Each focus group discussion lasted for 1 h and was conducted until saturation. The discussion was audio-recorded and the note taker also took notes. The recorded information was transcribed verbatim by a transcriptionist, and the transcribed work served as the basis of analysis for the study.

### Data analysis

Data were collected over a period of 3 months. Quantitative data were coded and entered into an Excel spreadsheet. Descriptive statistics of mean, standard deviation, frequency, percentage and range were used to summarise the data. Pearson correlation was used to test the relationship between scores on the knowledge of CVD and scores on the perception of CVD, and knowledge scores of CVD risk factors and perception scores of risk factors amongst participants. Independent *t*-test was used to test the difference in knowledge of CVD and its risk factors between male and female participants. One-way analysis of variance (ANOVA) was used to test the difference in knowledge of CVD and its risk factors across faculties. Level of significance for the quantitative aspect of the study was set at 0.05. Content thematic analysis was carried out on the data from the focus group discussion.

### Ethical considerations

Ethical approval was obtained for this study from the relevant authority and the reference number is UI/EC/18/0458.

## Results

A total of 433 copies of the questionnaires were distributed to the participants. However, 415 questionnaires were returned (response rate of 95.84%). Four hundred and two of the questionnaires (92.84%) were completed by the participants, and those questionnaires were deemed fit for analysis.

### Socio-demographic characteristics of participants

In all, 402 participants (59.5% female, 40.5% male) with a mean age of 20.94 ± 2.458 years participated in the cross-sectional survey aspect of the study (Table 1). The students were mostly between the age of 16 and 20 years and were distributed across the seven faculties (14.2% – 14.4%).

**TABLE 1:** Socio-demographic characteristics of the participants (*N* = 402).

Variables	Frequency (*n*)	Percentage (%)
**Age (years)**		
16–20	206	51.2
21–25	107	43.8
26–30	19	4.8
31–35	1	0.2
**Faculty**		
Art	57	14.2
Basic Medical Science	58	14.4
Law	57	14.2
Renewable Natural Resources	57	14.2
Science	58	14.4
Social Science	58	14.4
Veterinary Medicine	57	14.2
**Sex**		
Female	239	59.5
Male	163	40.5

### Participants’ knowledge of cardiovascular disease and its risk factors

Participants’ knowledge of CVD and its risk factors is shown in Tables 2 and 3. In Table 2, we observed that 39.1%, 35.1% and 25.9% of the participants had high, average and low levels of knowledge of the risks of CVD, respectively. The mean knowledge score of the participants was 15.31 ± 4.999. Table 4 revealed where these differences lie. The faculties of veterinary medicine and basic medical science had significantly higher scores than the other faculties. This table shows the faculty pair(s) that differed significantly after ANOVA testing.

**TABLE 2:** Knowledge level of participants.

Variables	Frequency (*n*)	Percentage (%)	
**Knowledge level**			
Low	104	25.9	-
Average	141	35.1	-
High	157	39.1	-
**Knowledge Score**			
Mean ± s.d.	15.31 ± 4.999	-	-
Minimum	-	1.00	-
Maximum	-	-	24.00

s.d., standard deviation.

**TABLE 3:** ANOVA showing distribution of participants’ mean level of knowledge score across faculties.

Faculties	Mean scores ± s.d.	*F* (df)	*p*
Art	12.65 ± 4.012	16.108 (401)	0.000
Basic Medical Science	17.90 ± 4.109	-	-
Law	15.21 ± 4.109	-	-
Renewable Natural Resources	14.02 ± 5.016	-	-
Science	14.10 ± 5.050	-	-
Social Science	14.00 ± 4.649	-	-
Veterinary Medicine	19.28 ± 2.477	-	-

ANOVA, analysis of variance; s.d., standard deviation.

ANOVA revealed significant differences in knowledge across the faculties, with faculties of veterinary medicine and arts scoring highest and lowest, respectively (*F* = 16.11; *p* < 0.01).

**TABLE 4:** Difference in mean knowledge level across faculties – Tukey HSD multiple comparison test.

Variable	Arts	BMS	Law	RNR	Science	Soc Sci	Vet Med
Arts	-	5.25*	2.56*	1.37	1.45	1.35	6.63*
BMS	−5.25*	-	−2.69*	−3.88*	−3.79*	−3.90*	1.38
Law	−2.56*	2.69*	-	−1.19	−1.11	−1.21	4.07*
RNR	−1.37	3.88*	1.19	-	0.09	−0.02	5.26*
Science	−1.45	3.79*	1.11	−0.09	-	−0.10	5.18*
Soc Sci	−1.35	3.90*	1.21	0.02	0.10	-	5.28*
Vet Med	−6.63*	−1.38	−4.07*	−5.26*	−5.18*	−5.28*	-

BMS, basic medical science; RNR, renewable natural resources; Soc Sci, social science; Vet Med, veterinary medicine.

* The mean difference is significant at 5%.

According to Table 5, smoking was the highest known (85.1%) risk factor of CVD amongst the participants, whilst diabetes was the least known (15.4%) risk factor. Physical activity, control of blood pressure, high cholesterol level and eating fatty foods were recognised by at least 80% of the participants as influencing the development of CVDs.

**TABLE 5:** Participants’ knowledge of specific cardiovascular disease risk factors (*N* = 402).

Statements	Correct		Incorrect	
	*n*	%	*n*	%
Smoking is a risk factor for heart disease (True)	342	85.1	60	14.9
Regular physical activity will lower a person’s chance of getting heart disease (True)	338	84.1	64	15.9
Keeping blood pressure under control will reduce a person’s risk for developing heart disease (True)	334	83.1	68	16.9
High cholesterol is a risk factor for developing heart disease (True)	332	82.6	70	17.4
Eating fatty foods does not affect blood cholesterol levels (False)	330	82.1	72	17.9
High blood pressure is a risk factor for heart disease (True)	304	75.6	98	24.4
If your bad cholesterol (LDL) is high, you are at risk for heart disease (True)	212	52.7	190	47.3
A person always knows when they have a heart disease (False)	199	49.5	203	50.5
If your good cholesterol (HDL) is high, you are at risk for heart disease (False)	156	38.8	246	61.2
People with diabetes rarely have high cholesterol (False)	124	30.8	278	69.2
People with diabetes tend to have low HDL (good cholesterol) (True)	90	22.4	312	77.6
Men with diabetes have a higher risk of heart disease than women with diabetes (False)	62	15.4	340	84.6

LDL, low-density lipoprotein; HDL, high-density lipoprotein.

Table 6 gives the distribution of participants’ perception across faculties. The mean perception score of the participants was 44.48 ± 6.557. Only 88 participants (21.9%) had a positive perception of CVD and its risk factors. The mean perception scores across the faculties were similar, with faculties of art and renewable natural resources having the best and poorest scores, respectively. Scores of male (45.52 ± 6.894) and female (43.77 ± 6.232) participants were also similar.

**TABLE 6:** Distribution of participants’ perception across faculties.

Variables	Positive		Negative		Mean scores ± s.d.
	Frequency (*n*)	Percentage (%)	Frequency (*n*)	Percentage (%)	
Art	20	35.1	37	64.9	45.84 ± 7.033
BMS	10	17.2	48	82.8	45.22 ± 5.161
Law	13	22.8	44	77.2	44.49 ± 6.431
RNR	10	17.5	47	82.5	43.09 ± 6.736
Science	13	22.4	45	77.6	43.62 ± 6.882
Soc Sci	11	19.0	47	81.0	44.40 ± 6.173
Vet Med	11	19.3	46	80.7	44.68 ± 7.236

BMS, basic medical science; RNR, renewable natural resources; Soc Sci, social science; Vet Med, veterinary medicine; s.d., standard deviation.

In Table 7, only 6.7% of the participants agreed that there was a possibility of them having a heart disease, whilst only 6.2% agreed that there was a possibility that they might have a heart disease in the future. Of the participants, 66.2% agreed that they were not doing anything unhealthy to their heart, whilst 36.5% of the participants agreed that people like them do not suffer heart disease.

**TABLE 7:** Overall distribution of participants’ responses to the perception of cardiovascular disease and its risk factors.

Statements	Strongly disagree		Disagree		Agree		Strongly agree	
	*n*	%	*n*	%	*n*	%	*n*	%
There is a possibility that I have heart disease	270	67.2	105	26.1	18	4.5	9	2.2
There is a good chance that I will get heart disease during the next 10 years	276	68.7	104	25.9	13	3.2	9	2.2
A person who gets heart disease has no chance of being cured	154	38.3	210	52.2	33	8.2	5	1.2
I have a high chance of getting heart disease because of my past behaviours	281	69.9	96	23.9	15	3.7	10	2.5
I feel sure that I will get heart disease	316	78.6	76	18.9	4	1.0	6	1.5
Healthy lifestyle habits are unattainable	217	54.0	147	36.6	30	7.5	8	2.0
It is likely that I will get heart disease	297	73.9	90	22.4	12	3.0	3	0.7
I am at risk for getting heart disease	282	70.1	92	22.9	21	5.2	7	1.7
It is possible that I will get heart disease	271	67.4	106	26.4	20	5.0	5	1.2
I am not doing anything now that is unhealthy to my heart	61	15.2	75	18.7	151	37.6	115	28.6
I am too young to have heart disease	83	20.6	139	34.6	104	25.9	76	18.9
People like me do not get heart disease	99	24.6	156	38.8	81	20.1	66	16.4
I am very healthy so my body can fight off heart disease	48	11.9	96	23.9	177	44.0	81	20.1
I am not worried that I might get heart disease	51	12.7	75	18.7	147	36.6	129	32.1
People my age are too young to get heart disease	116	28.9	168	41.8	72	17.9	46	11.4
People my age do not get heart disease	153	38.1	171	42.5	51	12.7	27	6.7
My lifestyle habits do not put me at risk for heart disease	43	10.7	64	15.9	174	43.3	121	30.1
No matter what I do, if I am going to get heart disease, I will get it	202	50.2	130	32.3	49	12.2	21	5.2
People who don’t get heart disease are just plain lucky	167	41.5	156	38.8	58	14.4	21	5.2
The causes of heart disease are unknown	198	49.3	152	37.8	39	9.7	13	3.2

### Association between knowledge and perception of cardiovascular disease and its risk factors amongst participants

There was no significant association between the knowledge and perception of the participants regarding CVD and its risk factors (χ^2^ = 2.506, *p* = 0.286; *r* = −0.038, *p* = 0.444).

### Perception of cardiovascular disease and its risk factors amongst undergraduates of selected faculties of the University of Ibadan: Exploratory qualitative study

Qualitative study to further explain and explore the perception of CVD and its risk factors using focus group discussions was conducted (Table 6). During the discussion, knowledge of CVD, perception of CVD, types of CVD, problems associated with CVD and prevention of CVD were explored. A group of undergraduate students participated in the focus group discussion. Sixteen students (four male, 12 female) participated in the focus group discussion. Two focus group discussions were conducted, with the first group consisting of six participants (three male, three female) and the second group consisting of 10 participants (one male, nine female). For the purpose of the discussion, participants were called by numbers: 1, 2, 3 till 10. Participants were asked questions in relation to the aim of the study. Four themes were generated.

#### Knowledge of cardiovascular disease

**Definition of cardiovascular disease:** Some participants defined CVDs as ‘the diseases of the heart’, some just acquired knowledge of it prior to joining the focus group discussion, whilst some came across it accidentally on the internet. Few of the participants had not known anything about CVD and its risk factors. This is as shown in the excerpts below (Table 8):

‘I know from what I read yesterday, it’s a disease of the heart.’

‘I’ve never heard about it. It just something I’ve seen somewhere, maybe online, it has to deal with the heart but I have limited knowledge.’ (Participant 4 FGD1, Female, Faculty of art)

‘I‘ve been hearing about this thing over the years and when you have anything to talk about cardio, it has to do with the heart; like cardiac arrest, it deals with heart attack and stuffs like that … The heart is like the central organ of the body and it connects everything, it’s just like you saying all your central port is faulty, every other part of the body will have its kind of shortcomings.’ (Participant 1, FGD1, female, faculty of art)

‘These are diseases related to the heart and blood vessels.’ (Participant 5, FGD1, Female, faculty of law)

‘I think they are diseases that are related to the heart.’ (Partcipant 10, FGD2, Male, Faculty of science)

‘Most times it can be congenital, it can be acquired, basically the acquired form is most times because of the things we do, mostly the things we eat, like cholesterol that can cause something like atherosclerosis.’ (Participant 6, FGD1, Male, Faculty of veterinary medicine)

**TABLE 8:** Themes and subthemes used for this study.

Number	Themes	Subthemes
1	Knowledge of cardiovascular disease	Definition of cardiovascular disease Risk factors of cardiovascular disease Examples of cardiovascular disease
2	Perception of cardiovascular disease risk factor	Individual perceived risk of developing cardiovascular disease Predisposition of young people to cardiovascular disease and its risk factor
3	Problems associated with cardiovascular disease	
4	Prevention of cardiovascular disease	

**Risk factors of cardiovascular disease:** Some of the participants had little knowledge of CVD risk factors. Many identified lifestyle, particularly diet and stress, as the risk factors. Some other risk factors mentioned were smoking, high blood pressure and physical inactivity. This is shown in the excerpts below:

‘Ideally one factor is what we eat and actually the previous speaker actually talked about cholesterol and I think it’s mostly contained in the fatty food we eat, processed food especially something like margarine. It has saturated fat in it. It affects our heart and the fact that we don’t exercise to burn the fat, these things can actually block the vessels of the heart.’ (Participant 5, FGD1, Female, Faculty of law)

‘I would say like lifestyle, because other than the fact that you eat, there’s a difference between eating and eating right and eating in the right proportion … again hereditary.’ (Participant 3, FGD1, Female, Faculty of basic medical science)

‘Talking about BP. High BP contributes to risk factor of having a cardiac arrest.’ (Participant 5, FGD1, Female, Faculty of law)

‘Physical inactivity, when you don’t walk, you just sit in one place and she also talked about dieting too.’ (Participant 5, FGD1, Female, Faculty of law)

‘My input is part of what they have said. Obesity, high blood pressure is related to cardiovascular disease. Fat accumulating in blood vessels that carries blood to and fro, then there would be much pressure on the heart.’ (Participant 5, FGD2, Female Faculty of clinical sciences)

‘I will like to say lack of exercises, taking of tobacco, and most of the cases I got to know that CVD are more in men than women.’ (Participant 6, FGD2, Male, Faculty of clinical sciences)

‘CVD can also be caused by stress, like if you stress yourself too much, you might end up having high blood pressure, some people are over thinking …’ (Participant 6, FGD2, Female, Faculty of art)

‘Things that are stressful, I will start with academics, 8-6 or 8-7 classes.’ (Participant 8, FGD2, Female, Faculty of art)

**Examples of cardiovascular disease:** Most of the participants did not know the diseases that comprise CVD. Some participants wrongly considered stroke as an example of CVD as shown in the excerpts below:

‘Hypertension’ (Participant 8, FGD2, Male, Basic medical science)

‘I think you can develop stroke’ (Participant 6, FGD2, Female, Faculty of art)

‘We have the likes of patent ductus arteriosus and ventricular septal defects.’ (Participant 1, FGD2, Male, Faculty of clinical sciences)

#### Perception of cardiovascular disease risk factors

**Individual perceived risk of devel oping cardiovascular disease:** Participants’ perception of the risk of developing CVD varied and is based on their understanding of CVD and its risk factors. Some individuals did not think they were at risk of developing CVD, whilst others said stress and poor diet could be the risk factors. This can be seen in the excerpts below:

‘I’ve never really thought of it, may be diet; like junks, I might have the risk factors but then I exercise.’ (Participant 8, FGD2, Male, Faculty of basic medical science)

‘I’ll say my diet too, because I eat a lot of fatty food.’ (Participant 6, FGD2, Female, Faculty of art)

‘I won’t say my lifestyle is exactly healthy but it doesn’t really reflect in my health, for example; I eat a lot of junk food, and I eat a lot but it doesn’t reflect my weight and everything. I don’t feel I’m at risk.’ (Participant 1, FGD2, Male, Faculty of clinical sciences)

‘I don’t think I have the risk, although there were sometimes that I actually had like a heart related issue, like all of a sudden my heart will just start beating fast and for some seconds I won’t be able to do anything, it will just be beating very fast and all of a sudden it’ll stop, so I don’t think I can say that one is even a cardiovascular disease.’ (Participant 9, FGD2, Female, Faculty of art)

‘When we talk of exercise, I exercise enough; I walk from my hall of residence to my faculty.’ (Participant 1, FGD2, Male, Faculty of social science)

‘No family history of hypertension, diabetes. Based on lifestyle I don’t do anything that’s dangerous to my health.’ (Participant 1, FGD2, Male, Faculty of clinical sciences)

‘The only thing I’ve noticed that affects me is stress and I try to rest a lot. No history of hypertension in the family.’ (Participant 2, FGD2, Female, Faculty of art)

**Predisposition of young people to cardiovascular disease and its risk factor:** Some participants opined that CVD can occur in young people, whilst some feel that only the risk factors could be seen in young people. This is as shown in the excerpts below:

‘I feel like with the lifestyle, yes you can actually develop it.’ (Participant 3, FGD2, Male, Faculty of basic medical science)

‘Yes, it could be seen in young people.’ (Participant 6, FGD2, Female, Faculty of art)

‘I actually think that young people are actually most times at risk.’ (Participant 3, FGD1, Female, Faculty of basic medical sciences)

#### Problems associated with cardiovascular disease

During the discussion, some participants felt that CVD could lead to loss of lives, some considered poor functioning of the heart and increased heart rate as problems associated with CVD, whilst some saw the psycho-social aspect of it, such as stress and hindrance in activities of daily living. This is seen in the excerpts below:

‘Most times we are always stressed and when you are stressed, it could lead to lack of sleep and then you have people having insomnia and bad eating habit for some people, number one is stress, then lack of sleep. The type of stress I’m saying is mental stress.’ (Participant 4, FGD1, Male, Faculty of art)

‘[*T*]he heart will not be performing its function usually, so most times, the person will get heart attack caused by the stress and then the person can have like high blood pressure and then all this like shortness of breath.’ (Participant 3, Male, Faculty of renewable natural resources)

‘It’ll lead to loss of live.’ (Participant 2, FGD1, Male, Faculty of social sciences)

‘One major factor is hypertension.’ (Participant 6, FGD1, Male, Faculty of vertenary medicine)

‘Increase in the heart rate.’ (Participant 1, FGD1, Female, Faculty of art)

Stroke; it’s actually something that is rampant in this part of the world.’ (Participant 1, FGD2, Male, Faculty of basic medical sciences)

‘We have the likes of exercise intolerance, poor blood circulation. Exercise intolerance is a condition whereby, should I say, the basic things one has to do he gets fatigued or fagged out easily, like someone walking from here to that door and is already gasping, so they don’t have the endurance to do what a normal person can do.’ (Participant 1, FGD2, Male, Faculty of clinical sciences)

‘I think it can hinder daily activities, also going in and out of the hospital might cost money.’ (Participant 1, FGD2, Male, Faculty of clinical sciences)

#### Prevention of cardiovascular disease

Some participants mentioned strategies that the university can put in place to help them reduce CVD risk factor, such as stress-free curriculum and health guides for students. Some participants talked about improving the financial capability of students, reducing the cost of food and making food easily available. This is shown in the excerpts below:

‘Our number one approach will be the poverty aspect … The effect of financial capability on health status in students … a form of poverty alleviation.’ (Participant 6, FGD1, Male, Faculty of vertenary medicine)

‘I’ll suggest to drink a lot of water will be number one. Number two, I think the school management should try and improve their welfare and departments should have avenue to have someone to talk about their problems to. School calendar should create space, so people have time to themselves, when the school is in session, maybe on Fridays-lecture free and Saturdays too.’ (Participant 5, FGD1, Female, Faculty of law)

‘Concerning stress, we’ll try and rest, get rest. Manage your weekends well.’ (Participant 2, FGD2, Female, Faculty of art)

‘I feel like there should be something like the bulletin or something that should be released, like a health guide that tells people what to do like an example these are fruits you can eat, this is vegetable you can eat and it should be something they should be doing regularly or weekly so as to create awareness to students and all. And I also feel like there should be regular check-up, I don’t know if it’s possible like every session, like doctors or nutritionist goes to halls of residence and do like an awareness and then there’s check-up for students.’ (Participant 3, FGD1, Female, Faculty of art)

‘I believe there should also be an education on that whereby parents, especially the onset of pregnancy, they’ll go for proper check-up and to know what their child is predisposed to and they will be able to take appropriate measures.’ (Participant 6, FGD1, Male, Faculty of Clinical Sciences)

‘I think if food can be more affordable for students, it’ll prevent people from eating junks.’ (Participant 2, FGD2, Male, Faculty of clinical sciences)

‘Diet, eating healthy can prevent it, if the person doesn’t take tobacco, reduce alcohol intake and the likes.’ (Participant 3, FGD2, Female, Faculty of art)

‘I feel like proper education of the proper way of life, the kind of life I’d live is not the kind of life a very fat person should live, so, people should look at their family and know the medical history they have and know how to take care of themselves, they should be informed.’ (Participant 3, FGD2, Female, Faculty of art)

## Discussion

### Knowledge of cardiovascular disease and its risk factors

Findings from the study showed that overall knowledge level of CVD and its risk factors was poor. Majority of the participants did not have adequate knowledge. The findings are similar to a study on undergraduate students in Palestine, where they also had a fair level of knowledge.^16^ The level of knowledge of the participants in the present study is higher than that observed in a previous study of Nigerian older adult university workers^8^ but lower than the result amongst medical students in the United Arab Emirate (UAE).^17^ The fact that participants of the present study were recruited from different faculties as opposed to those of the UAE study, who were all medical students, could have resulted in a better knowledge outcome in the latter.

From the qualitative study it was observed that participants did not have good knowledge of CVD and its risk factors, thus buttressing the findings from the quantitative component of the study. Some of the participants could describe CVD as a disease of the heart, with only a few describing it as both a disease of the heart and blood vessel. This is similar to another report.^21^ Participants were, however, seen to have good knowledge about smoking, low physical activity, high blood pressure, unhealthy diet, obesity and stress as risk factors of CVD, although they were not able to elucidate how these factors contribute to CVD. This is consistent with previous findings,^22,23,24,25,26^ where participants were unable to link lifestyle risk factors to CVD. The high level of public sensitisation on these factors could be responsible for this. However, participants displayed poor knowledge about diabetes and cholesterol as a risk factor of CVD, similar to previous reports.^8,25^ Inasmuch as the participants acknowledged high cholesterol as a risk factor of CVD, they could not differentiate the roles played by good and bad cholesterol. Participants of similar previous studies also exhibited difficulties in differentiating the role of good and bad cholesterol in the development of CVD.^8,23,24^ Similar to previous reports,^8,27^ participants of the present study wrongly posited that a sufferer of CVD will always be aware of his/her disease condition. Generally, participants of the present study did not know the mechanisms through which explored risk factors contribute to CVD development. This is not surprising as most of the participants were not medically inclined.

### Perception of cardiovascular disease and its risk factors

Our findings showed that participants from this study had a negative perception of the risk of developing heart disease. They felt that they could not have CVD irrespective of their individual lifestyle but other people could have CVD. This is similar to a study conducted amongst female university students in South Africa^28^ and medical students in the UAE,^17^ where majority of the participants did not consider themselves as being at risk of developing CVD. This result is not surprising in a country like Nigeria with numerous cultural and religious beliefs, where people do not want to be associated with negative situations such as diseases, although they might have it.

A negative perception of being at risk of CVD was further confirmed by the outcome of the qualitative study, where a majority of the participants categorically denied having a risk of heart disease despite their lifestyle risk factors, never believed they could develop heart disease in the future and denied doing anything that would endanger their cardiovascular system. Some participants even claimed to be exercising enough, whilst further probing revealed that their idea of exercise was wrong. Some participants who acknowledged indulging in unhealthy lifestyles like stress and unhealthy eating still denied having a chance of developing CVD. Another study of African American participants equally reported that stress, unhealthy diet and inadequate physical activity were perceived as CVD risk factors.^26^ The participants equally opined that people like them, in terms of age and physique, could not suffer from heart disease. However, similar to another report,^29^ participants believed that healthy lifestyles for controlling CVD development are attainable depending on how an individual grew up.

### Difference in knowledge and perception of cardiovascular disease and its risk factors across gender and faculties

The findings of this study revealed no significant difference in the knowledge and perception of CVD and its risk factors between male and female participants. This is in contrast to previous Iranian and Singaporean studies,^30,31,32,33^ where females had significantly better knowledge than their male counterparts. Current literature seems to have few data on sex differences in the knowledge of CVD and its risk factors.

We observed a significant difference across faculties in the knowledge of CVD and its risk factors. Participants from faculties of veterinary medicine and basic medical science had a majority of students with high knowledge level. This is logical as these two faculties are related to medicine and might have exposed their undergraduates to knowledge about CVD risk factors during the course of their training. This result was further highlighted by the qualitative aspect of the study. The impact of medical exposure on the knowledge and perception of CVD can be seen in the result of a previous Croatian study,^34^ where freshmen medical students had significantly lower knowledge about CVD than their final year counterparts. There has also been reports of good and poor knowledge amongst medical and non-medical students, respectively.^17,35^

### Prevention of cardiovascular disease and problems associated with it

Results of the qualitative study highlighted health, social and financial issues as the problems CVD sufferers face. As it relates to the prevention of CVD, most of the participants dwelt on specific preventive measures that the university could employ rather than general preventive measures against CVD. The participants strongly believed that the university can help them with their lifestyle choices that put them at risk by addressing some of the challenges that increase unhealthy lifestyle choices. Findings from a previous study show that addressing financial issues helps prevent bad lifestyle choices that are risk factors for developing CVD.^22^ From the information the participants provided on prevention of CVD, it could be observed that most participants had a mindset that accentuated dependence on the university authority is necessary for a change in lifestyle choices, with only a few believing that they could change their wrong lifestyles on their own. This response was similar to the preventive measures stated in a study of African Americans.^26^

## Limitation

Recall bias is associated with questionnaire study as participants may not be able to remember things accurately. However, this study was about knowledge Therefore, this limitation did not pose a serious problem. Besides this, the introduction of the qualitative aspect could have attenuated the problem of recall bias. The outcome of this study provided insights into the level of knowledge and perception of CVD and its risk factors amongst adolescents and young adults in a university setting. This will be useful in identifying any further need for CVD prevention programmes in the setting of this study, thereby reducing possible risk factors and the likelihood of occurrence of CVDs in future.

## Conclusion

Undergraduates of the University of Ibadan had a fair knowledge and poor perception about CVD and its risk factors. Their knowledge and perception of CVD did significantly associate with each other and did not significantly vary across gender. Students from medical-related faculties had better knowledge and perception than others. There is a need to improve the knowledge of CVD and its risk factors amongst university undergraduates. This can be in the form of improving public awareness about CVD or inclusion of CVD and its risk factors as a topic in the general study course for non-medical undergraduates. Qualitative study identified learning environment as a major determinant in adopting healthy lifestyle habits such as recreation facilities and time for exercise during school time. There is therefore a need for the university to put in place a more conducive learning environment for students to adopt healthy lifestyles.
